# Indoor tent management for extending honey bee research season: benefits and caveats

**DOI:** 10.1093/jisesa/iead113

**Published:** 2024-05-28

**Authors:** Trevor Bawden, Adam G Dolezal, Chelsea N Cook

**Affiliations:** Department of Biological Sciences, Wehr Life Sciences, Marquette University, Milwaukee, WI, USA; Department of Entomology, University of Illinois Urbana–Champaign, Urbana, IL, USA; Department of Biological Sciences, Wehr Life Sciences, Marquette University, Milwaukee, WI, USA

**Keywords:** honey bee, behavior, ecology and behavior

## Abstract

Honey bees are important organisms for research in many fields, including physiology, behavior, and ecology. Honey bee colonies are relatively easy and affordable to procure, manage, and replace. However, some difficulties still exist in honey bee research, specifically that honey bee colonies have a distinct seasonality, especially in temperate regions. Honey bee colonies transition from a large society in which workers have a strict temporal division of labor in the summer, to a group of behaviorally flexible workers who manage the colony over winter. Furthermore, opening colonies or collecting bees when they are outside has the potential to harm the colony because of the disruption in thermoregulation. Here, we present a simple and affordable indoor management method utilizing a mylar tent and controlled environmental conditions that allows bees to freely fly without access to outdoor space. This technique permits research labs to successfully keep several colonies persistently active during winter at higher latitudes. Having an extended research period is particularly important for training students, allowing preliminary experiments to be performed, and developing methods. However, we find distinct behavioral differences in honey bees managed in this situation. Specifically learning and thermoregulatory behaviors were diminished in the bees managed in the tent. Therefore, we recommend caution in utilizing these winter bees for full experiments until more is known. Overall, this method expands the research potential on honey bees, and calls attention to the additional research that is needed to understand how indoor management might affect honey bees.

## Introduction

Honey bees are excellent organisms for research and teaching. Honey bees are important to study in many fields: they are economically valuable (Chopra et al. 2015); they are a model organism for genetics, physiology, and behavior; their complex social dynamics inspire the analysis of distributed organization in engineered systems; they play important roles in ecosystems; and their haplodiploid mating system and eusocial societies are evolutionarily interesting. Colonies are easy and affordable to procure; most regional honey bee clubs sell packages for $150–200 in the United States (in 2023). If colonies are well cared for (fed, treated for diseases, especially for varroa mites, and prepared effectively for winter), they can last several years. These factors together make honey bees ideal organisms to study in research labs as well as useful model animals to potentially use in college classrooms with supervision and safety protocols. However, one major hurdle complicates the use of honey bees in research and teaching: their inactivity for several months, which largely overlaps with classes and student availability. Here, we aim to outline an affordable and easy method for extending the use of honey bee colonies during high-latitude winters by managing them inside tents indoors.

In temperate regions in the northern hemisphere, temperature and precipitation can prevent access to bees from occurring from October to May. To survive the winter, honey bees must effectively manage the temperature and humidity inside their colony. This is done by clustering and shivering (Omholt 1987, Stabentheiner et al. 2003). The cluster will expand and contract as ambient temperature changes (Southwick 1983, Sumpter and Broomhead 2000, Ocko and Mahadevan 2014). The queen and the brood will remain at the center of the cluster (Fahrenholz et al. 1989). However, no brood is reared from about November through January in the colder regions in the northern hemisphere (Winston 1991). This clustering behavior is critical to maintaining warm colony temperatures during cold ambient temperatures (Stabentheiner et al. 2003, Ocko and Mahadevan 2014), thus disrupting this cluster can lead to colony death. The importance of the winter cluster limits collecting abilities for research during winter months.

In addition to seasonal weather patterns preventing access, honey bee physiology and behavior dramatically changes. During the summer, workers change jobs over their lifetime, transitioning from nursing to peripheral hive tasks, to foraging as they age (Oster and Wilson 1978, Winston 1991). Winter bees are “generalists” and perform tasks as needed, not related to their age (Winston 1991). Honey bees also do not seem to perform typical colony tasks, like fanning when experiencing increasing temperatures in the lab (Cook et al. 2016). Winter “diutinus” bees live for several months, as opposed to the typical summer worker honey bee lifespan of 40–50 days.

Several environmental and physiological factors drive the transition from summer to winter bees. The reduction of food available, temperature, and day length likely all cue this shift (Döke et al. 2015). Reduced pollen collection triggers a reduction in brood production (Mattila and Otis 2007). Shortened day length caused a major colony shift in behavior, including increased food consumption and increased fatty tissue in individual bees (Fluri and Bogdanov 1987). A drop in temperature results in a decrease of juvenile hormone (Huang and Robinson 1995) and affects other downstream factors like vitellogenin (Fluri et al. 1982). Because these physiological and behavioral differences may disrupt experiments even in colonies kept in stable, room temperature conditions, care must be taken in choosing uses for bees derived from these colonies and interpreting data from these studies.

Extending the period that honey bees can be used has extensive benefits beyond conducting research. Winter honey bee workers can be used in training new students in basic techniques, such as safety, collecting, and assays used in the lab. Graduate students or continuing undergraduate researchers can use indoor bees to develop protocols and test methods. Furthermore, much of the bee season does not overlap with the semester university schedule. As such, much of the opportunity to use bees in the classroom or as outreach is lost.

Research programs in temperate regions that utilize honey bees would benefit from extending their season. Keeping bees indoors to increase research, teaching, and outreach is a perennial goal of honey bee researchers (Pernal and Currie 2001). Scientists studying the impacts of circadian rhythms and other chronobiological features designed rearing and flight rooms to control for environmental conditions. These methods focus on providing bees with a dedicated space that mimicked outdoor conditions like sunlight so that extensive research could be conducted on the colonies (Poppy and Williams 1999). In fact, some of these methods are able to keep colonies inside for years (Renner 1955, Jay 1964). The designs of these spaces provide sophisticated methods to continue conducting research on honey bees in tightly managed, natural conditions. However, many researchers have limited indoor space, and cannot devote resources to indoor experiments.

Here, we aim to provide a simple, affordable method to extend the research season of honey bees that does not require construction or devotion of an entire room to indoor wintering. This technique has a relatively small space requirement and uses basic items easily found at most hardware stores or online. Although this extends our training period, we explored the hypothesis that honey bees from colonies kept inside exhibit behavior that is different from what is expected from bees outside during the summer. As such, we found behavioral differences in 2 behavioral assays: learning and fanning. Overall, this method allows the honey bee season to extend for many benefits, but we caution users on relying on behavioral results during this period until further is known about winter bee behavior.

## Experimental Design

### Room and Tent Choice

We chose a room that did not have windows so that we could control the day/night cycle of the bees. This prevents bees from orienting to windows to try to escape. It also aids in environmental regulation; during the winter, windows tend to make rooms colder. This room is an internal room in our lab and houses an incubator, refrigerator, and freezer, as well as a cabinet of tools. We did have to modify the door to the room, which included foam door jam insulation and rubber weather stripping (Home Depot) to prevent light from the main lab from shining in, as well as to prevent any escaped bees from orienting toward the light and escaping into the main lab. An ideal room will have no external light exposure as bees will orient toward windows.

**Fig. 1.**
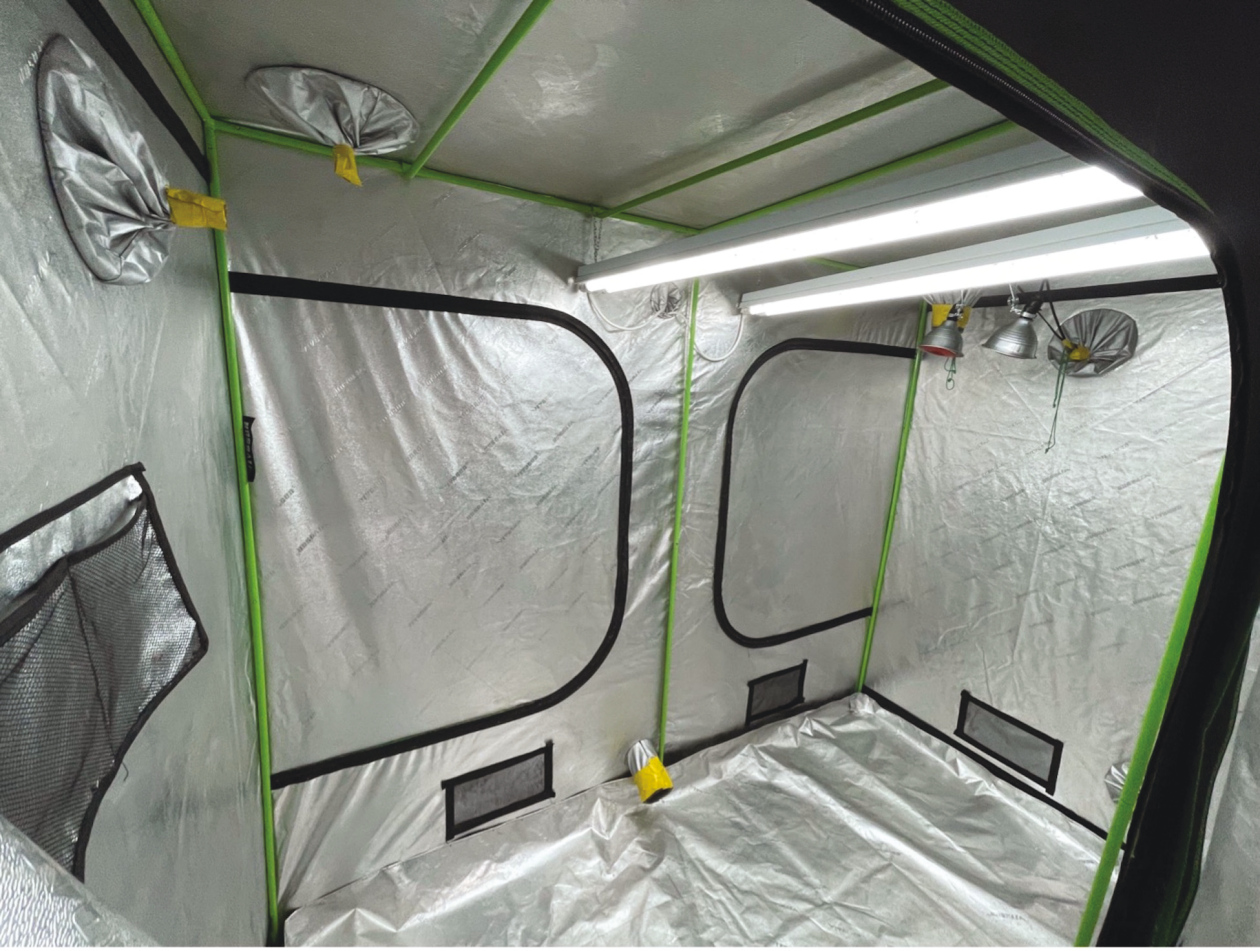
A photograph of the tent after colonies were removed and tent was cleaned. Photograph by Elsa Hahn.

**Fig. 2.**
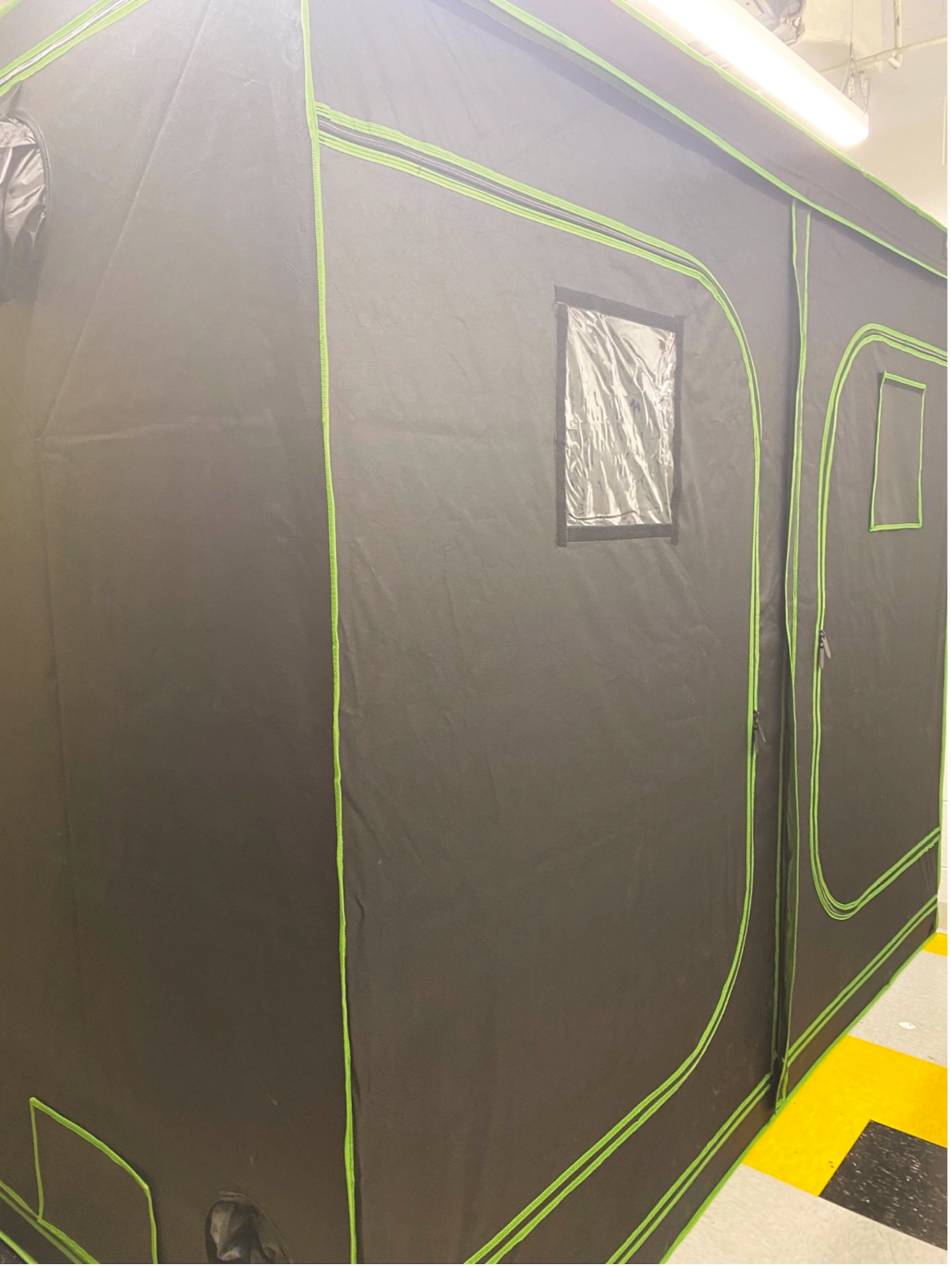
An outside view of the tent with left window cover removed, right window cover in place. Photograph by Justine Nguyen.

As the selected room was multi-use, we decided the bees required secondary containment. Many types of enclosures are possible. We found the mylar grow tent was affordable, came in many sizes, easy to put up and take down, easy to store when not in use, and easy to clean. These tents are typically utilized for indoor plant grow rooms. We chose a tent by brand Vivosun that was 2.4 m height, 1.2 m width, 2 m long. It is sealed on all sides except for the exhaust and zipper doors. Both width sides and ceiling had circular 0.3 m diameter exhaust/electrical ports. If we didn’t use them, we sealed them with duct tape (Fig. 1). Both length-sides of the tent had 2 zipper doors. One door had a 0.3 m × 0.3 m soft plastic window that came with a Velcro cover. It has a powder coated structural metal frame. There is an additional bottom cover to protect the frame. The tent allowed for effective containment of the bees, as well as a limitation of impact on the rest of the room, such as smell, feces, and dead bees. The tent does not allow external light in unless the window cover is removed (Fig. 2), which prevents the overhead room lights from impacting the bees. However, many types of tents could work, such as a mesh tent. For some rooms, a custom tent might be necessary.

### Summer and Fall Preparation

We fed colonies as needed, including a 60% sucrose syrup or commercially available high fructose corn syrup and commercially available pollen patties (Mann Lake). In October, we prepared 2 nucleus colonies to go into the tent. We aimed for very healthy, robust nucleus colonies to account for population losses that will occur all winter, which we characterized as 5 frames of bees as observed from above inside a 5-frame nucleus box and weighing approximately 18 kg. Each colony was tested and managed for parasites and pathogens, especially *Varroa destructor* mites, as the colonies will still rear brood while inside, making them especially susceptible. (Rademacher and Harz 2006).

## Procedure

### Moving Into Tent

To move the colonies into the tent, we waited until winter bee production stopped and the colony population was stable, which was identified by inspecting the colony for brood. When there was no more brood production, we chose a cold day, ideally below 7 °C which eliminated foraging flights, so the maximum number of bees were brought inside. We prefer daytime movement for safety and aim for early morning or late afternoon to avoid other people who may be in the building. This occurred 18 November 2022 in Milwaukee, WI. However, this date may differ at different latitudes and different years, so we urge flexibility on this date (Mattila et al. 2001). For initial feeding, we purchased commercial pollen and used a spice grinder (Table 1) to grind into a fine powder, then pressed 100 g of ground pollen into the cells of a drawn comb frame, taking care to not damage the cells. We did this by carefully dragging handfuls of pollen powder across the cells and smoothing it in until cells were half full. This pollen supplementation is critical because as food availability in the environment decreases, and the summer bees die off, very little additional pollen comes into the colony, and bees will feed on this during fall dearth. Pollen substitute was not enough for complete brood production in the tent (meaning the queen laid eggs but they did not develop into pupae), so we emphasize the use of real pollen during this time. The mylar tent was set up in a small windowless lab room (3.5 m × 3.2 m; Fig. 1). All equipment is listed in Table 1.

**Table 1.** Material List

Item	Brand name	Description	Use
Mylar Grow Tent, 96ʹ × 48ʹ × 80″	Vivosun	Hydroponic grow tent	Bee enclosure
Two LED red lights	Sylvania 60-Watt Equivalent PAR38 Red LED Light Bulb	Red lights	Light so humans can see, but the bees cannot
White LED lights	WYZM	120 V, 7 W, 4200 Lumens, 5500 K Shop Lights	Mimics the sun in the tent
Two 24 h Timers for lights	Smart Electrician 15 AMP outlet timer	Light timer	Adjusting the duration of light bees are exposed to
Exhaust fan	AC Infinity RAXIAL S4, Inline Booster Duct Fan 4” with Speed Controller	2 duct fans, 1 for air intake, 1 for exhaust. 106 CFM airflow.	Air intake/exhaust for the tent
Carbon filter	Vanleno 4inch Carbon Filter Odor Control	Carbon filter that attaches to outflow duct.	Odor control, allergen minimization.
Paper	USG Fiberock brand	Industrial paper	Covers the tent floor for easy cleaning
Hive stand	2ʹD × 4ʹL × 2ʹW	Wood rack	Keeps colonies at ergonomic height
Glass jar	Ball canning pint jars with lid	Glass Jar with lid with silicone nipple attachment	Feeding bees sucrose
Bleach	Arocep Ultra Bleach	1/3C of bleach per 1 gallon of warm water (per instructions on bottle).	Sterilizing tent
Sugar	Great value Walmart	White crystalline sugar, 1:1 sugar and water by weight.	Food–carbohydrates
Pollen	Patz Maple and Honey Farm pollen whole grain 5 lb	Feeding bees	food–protein
Coffee grinder	Wancle Brand Electric Coffee/Spice Grinder	Breaking down pollen to a fine powder. About 1 minute of grinding for a full grinder.	Food preparation

### Indoor Environmental Management

**Fig. 3.**
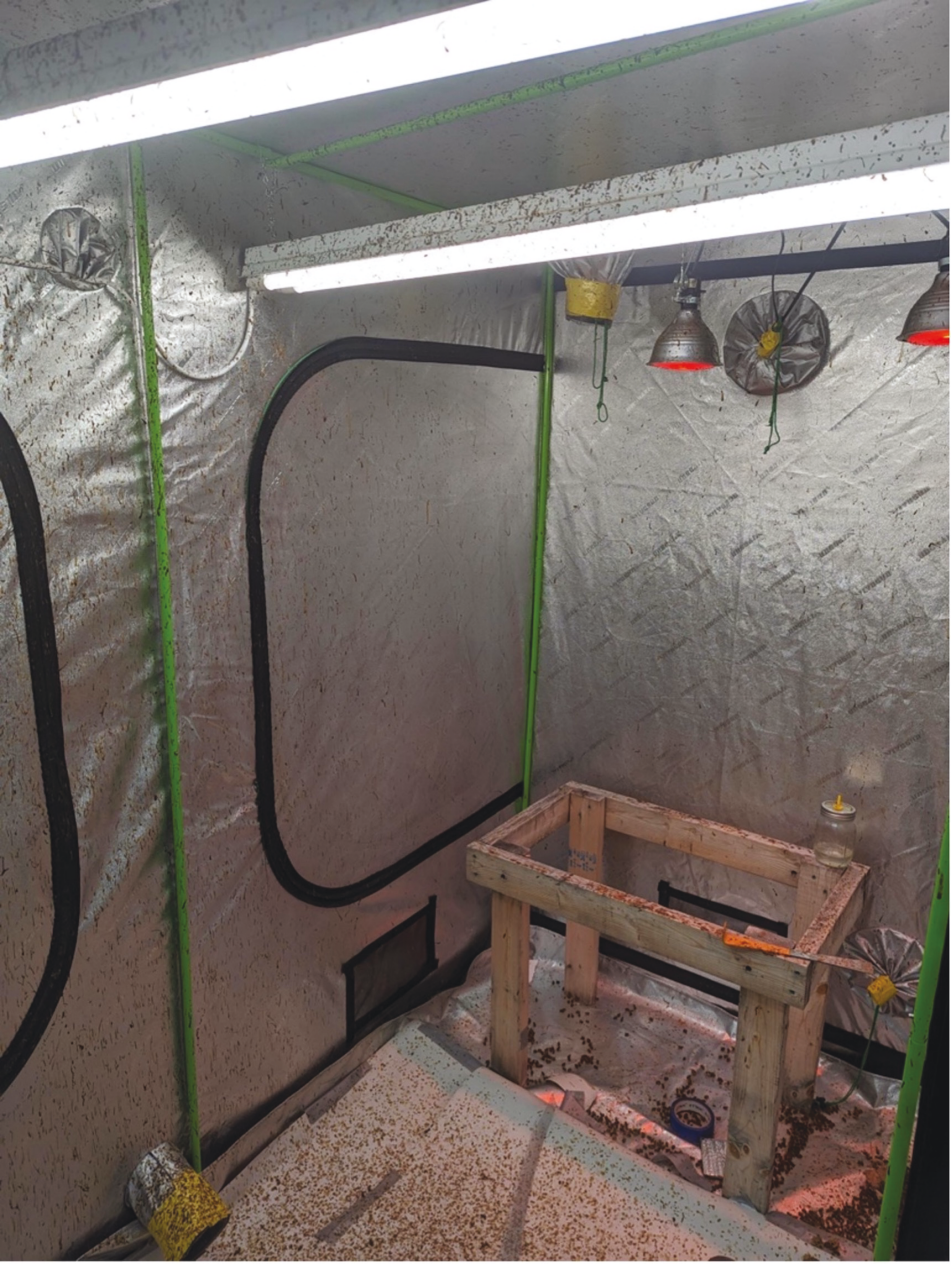
A photograph of the inside setup of the tent with bees removed. Photograph by Trevor Bawden.

The tent we used provides enough room for 3 five-frame nucleus colonies (Fig. 3); Table 1 lists the tent information and details on the materials used for environmental control. Temperature was managed at typical room temperature, 25 °C with no additional heating or cooling aside from building HVAC. We set day length to a 16H daylight cycle controlled by outlet timers. We used broad spectrum, white LED lighting which was the same as the rest of the lab. Bees did cluster on the lights and on the colony, which did not seem to negatively impact them as foraging flights occurred even in the small space. Red light was used when bees needed to be collected, but during the bee’s nighttime circadian rhythm. We provided airflow, which included an intake fan and an exhaust fan. The exhaust fan was fitted with a carbon filter to reduce odor. About 2 nucleus colonies typically consumed 215 g of 50% concentration of sucrose syrup per week and 100–200 g amount of pollen every 3–4 wk. Sucrose was provided in a pint mason jar nipple feeder, and pollen patty substitute was placed on top of the colony’s frames or dried pollen was rubbed into empty drawn comb. Food was replenished every week, and food containers were cleaned. Bees will defecate and die, so the tent needs to be cleaned every month. This includes the removal of dead bees, changing the paper lining the floor, and spot cleaning the walls and ceiling with water, bleach solution (per instructions on the bottle), and a sponge. Most of the bee losses will occur in the first month, as the last of the summer bees die off. We weighed the dead bees weekly, and the average weekly weight of all the dead bees from both colonies was 65.44 g.

### Safety

We maintained typical beekeeping safety while colonies were inside. Full bee suits were worn while inside the tent. The tent was placed inside a room with no windows, which was inside the lab’s designated space. Doors were locked when the lab was not occupied. Signs were placed on the door alerting facilities and custodial staff to the presence of bees inside. We sealed the door with insulation and place a door sweep along the bottom of the door. This provided 2 benefits: minimizing light from the lab and preventing any crawling bees from crawling under the door. When opening the tent, the lights of the room were kept off so that bees would not fly out of the tent. When entering the tent, a sign was placed on the door alerting others to not enter the room at that time.

### Transition Back Outside

We transitioned the 2 nucleus colonies at the end of March on a >10 °C day. We combined the 2 nucleus colonies together using the newspaper method (Caron and Connor 1999) and returned to the bee yard. One queen was used in another colony. The colonies effectively transitioned and survived.

### Behavioral Impacts of Indoor Tent Wintering

**Fig. 4.**
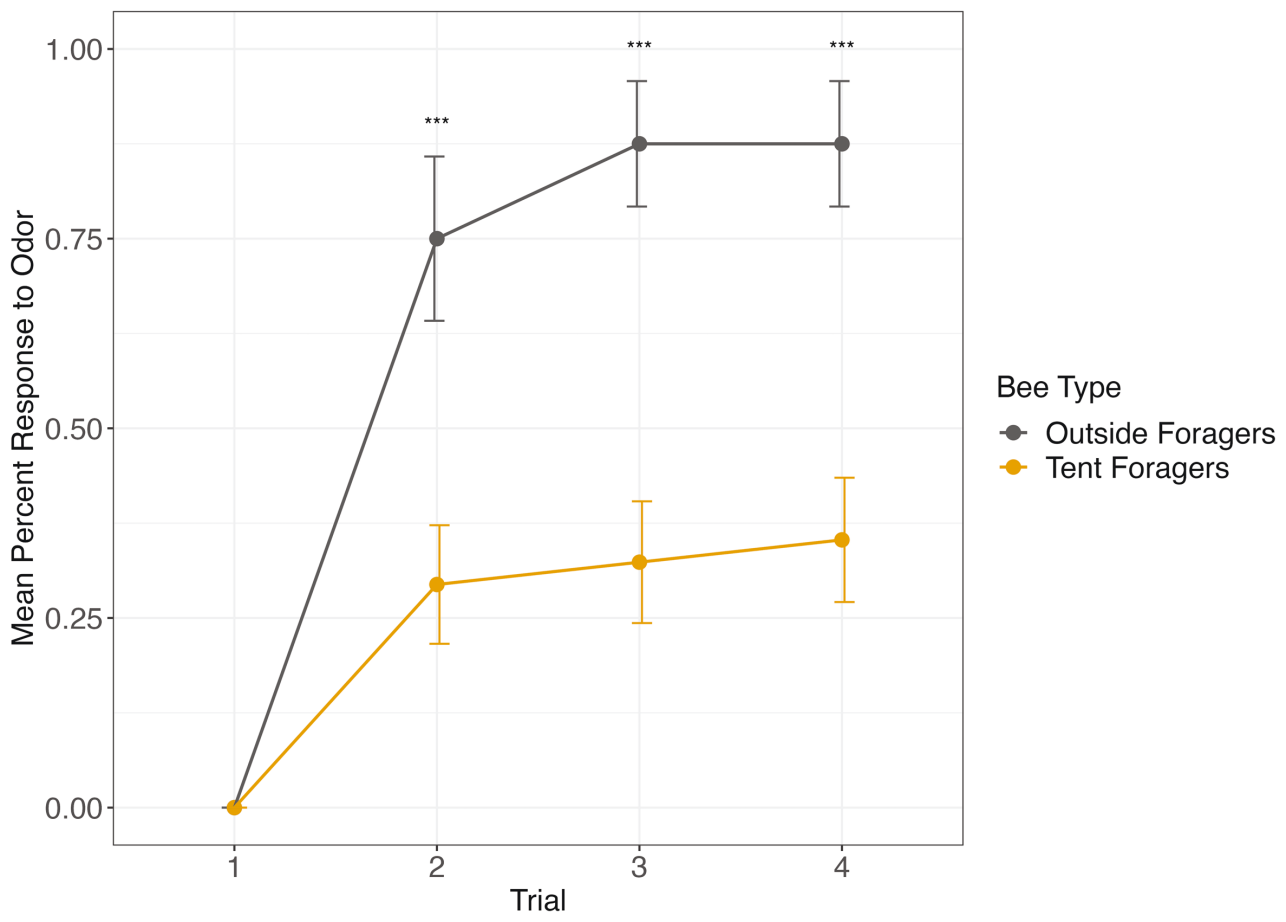
Indoor tent bees learn significantly less compared to foragers collected outside during an olfactory classical learning assay. As trials progressed, forager bees collected outside learned significantly more than bees collected inside the tent. noutside = 16, ntent = 34. Error bars represent standard error. Asterisks indicate significance at *P* < 0.001Tukey post hoc pairwise test.

**Fig. 5.**
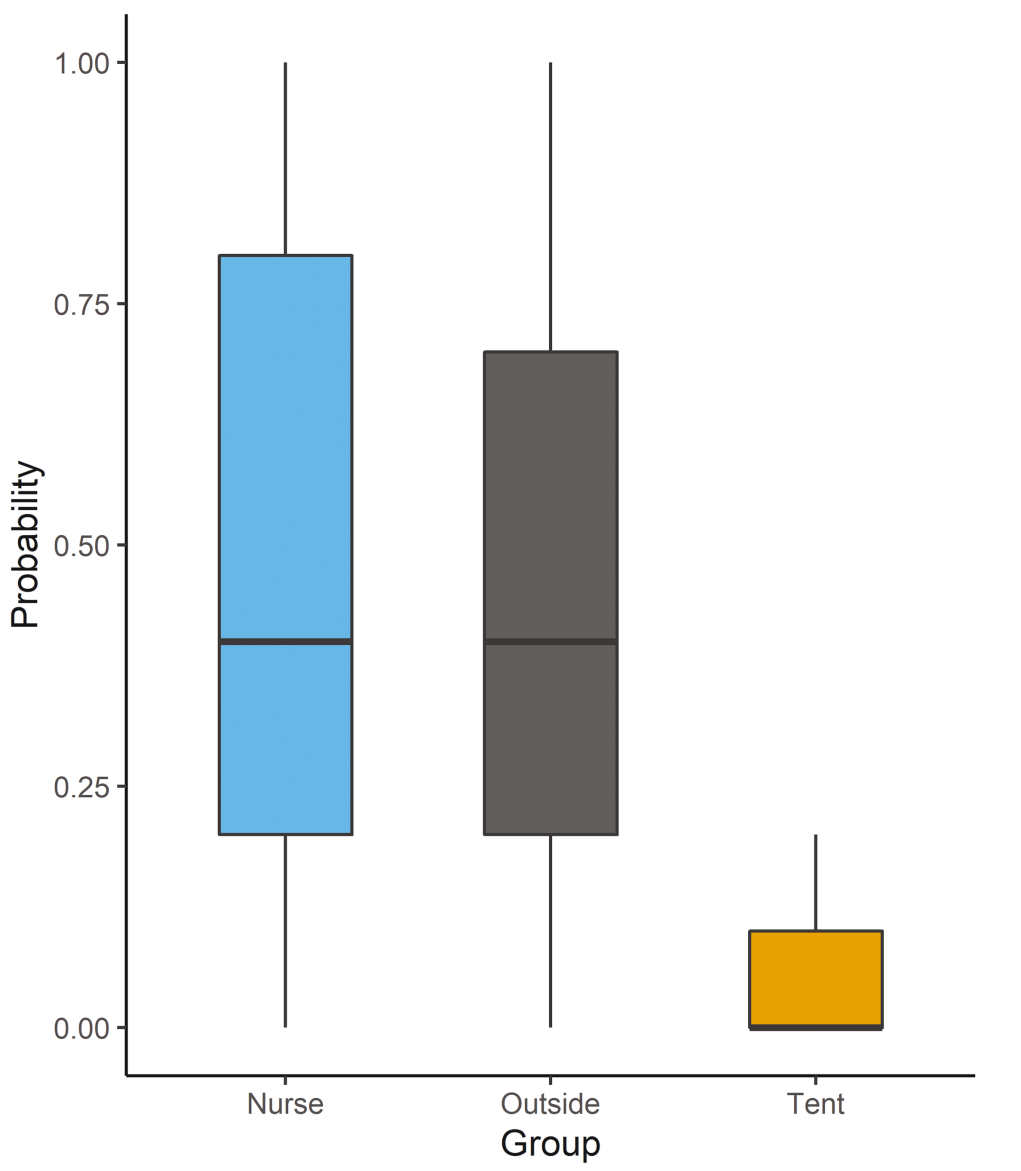
Bees collected from the indoor management tent were significantly less likely to fan compared to fanners and nurses collected from summer colonies. We collected bees and tested their fanning behavior (Cook and Breed 2013). Data were collected across several years (Nurses in 2015, *n* = 29; outside fanner bees in 2022, *n* = 15; and indoor tent bees in 2022, *n* = 15). Data are depicted as boxplots, where black lines are medians, boxes represent 25%–75% of data, and whiskers represent 95% of the data. Letters indicate significance of *P* < 0.05 based on Tukey post hoc test.

While using these colonies, we were able to collect data on the performance of these bees during typical behavioral assays used in our lab that illustrate the behavioral impacts of indoor management. We performed 2 assays: the proboscis extension reflex to evaluate learning behavior (Fig. 4), and a fanning assay to evaluate the likelihood of performing the thermoregulatory fanning response (Fig. 5). These data are for illustrative purposes only and were not used to test explicit hypotheses about tent bees compared to outdoor bees. Overall, keeping bees inside appears to alter their behavior significantly. As such, we caution the use of indoor bees for behavioral experiments without sufficient controls.

#### Methods for behavioral testing

##### Assessing honey bee learning using the conditioned proboscis extension reflex

To evaluate the learning capabilities of honey bees, we performed a typical classical conditioning assay using the proboscis extension reflex (Takeda 1961, Bitterman et al. 1983, Smith and Burden 2014). We did this at 2 different times to compare summer colonies that were outside and colonies that were wintering inside. We maintained a rooftop apiary of 6–8 colonies on the second-floor roof of Wehr Life Sciences on Marquette University’s campus. To collect forager honey bees during July–August 2022, we placed 1/8″ hardware cloth at the entrance of the typically maintained colony to prevent foragers from entering the hive. Pollen foragers were identified as returning bees with pollen in their corbiculae. We then used soft forceps to collect bees and place them into glass scintillation vials with ventilation holes drilled in the top. To collect bees in the tent during November–December 2022, we used soft forceps to collect individual bees that were out of the colony, either foraging for food or had been seen landing on a surface out of the colony. Bees were then placed in glass scintillation vials which had ventilation holes. Once collected, we placed bees on ice until immobilization, about 3 min. We then placed bees into metal harnesses and used duct tape secure them, with one piece (10 cm × 2 mm) between the head and thorax and one (10 cm × 2 mm) around the abdomen. Once bees woke up, they were fed 1 M sucrose solution and allowed to acclimate for 1 h. We then tested for sucrose responsiveness to 1 M sucrose. If they did not respond, they were not used in the assay. During acclimation, odor tubes were prepped by placing 3 ml of octanone (Sigma Aldrich) onto a strip of filter paper (10 cm × 5 mm), and then placing the filter paper into a glass syringe. The syringe was capped with a rubber stopper with a hole to allow for air to flow through. To perform the assay, we connected the odor tube to tubing where air could be directed through the odor tube to disperse the air. The tube was then positioned in a plexiglass arena to direct airflow toward the antennas of the honey bee. A bee was then placed into the plexiglass arena for training. A typical training bout lasted about one minute: 24 s for acclamation, 5 s of odor exposure with 1 second of 0.2 ml of 1.5 M sucrose touched to the antennae then to the proboscis overlapping with the last second of odor exposure, then 25 s of rest before moving the bee out of the arena a placing the next bee in for training. Learning was quantified as the bee extending her proboscis within the first 4 s of odor exposure, before the sucrose was presented. For a thorough PER method, see (Smith and Burden 2014). We performed a logistic regression on learning responses. All statistical analyses were performed in R (v4.2.2) and RStudio (v2023.03.0 + 386).

##### Assessing honey bee thermoregulatory fanning response

To test fanning, we performed a typical fanning assay on 3 different task groups: fanners, nurses, and tent bees. We used 2 different data sets for this comparison: one of fanners and nurses from 2015, and tent bees from 2022. To collect fanners and nurses during the summer of 2015, we used an apiary with 8–10 colonies maintained at the University of Colorado, Boulder. We identified fanning bees at the entrance of a colony by their fanning wings, remaining still other than fanning wings for 30 s, and a curved abdomen (Cook and Breed 2013, Egley and Breed 2013). We collected bees into mesh cages (cylinder: 4.4 cm × 13.4 cm) and placed them in groups of 5. To collect nurses, we opened the colony and removed a brood frame. We then identified nurses as bees who were on the brood frame either tending larvae or cleaning cells (Seeley and Kolmes 1991, Kaspar et al. 2018). We collected them using soft forceps and placed them into mesh cages in groups of 5. In the tent, there were rarely fanner bees as the temperature in the room was maintained at 25 °C. To collect bees from the indoor management tent, we used soft forceps to collect bees from the entrance of the colony. We placed them into mesh cages into groups of 5. None of the bees collected from the tent were actively fanning. We then brought caged bees into the lab and placed them into the heating apparatus for a 25-minute acclimation. To test the fanning response, we heated bees 1 °C/minute and recorded the number of fanners and the temperature at which they began to fan. We performed a logistic regression on number of fanners, and a linear regression on fanning temperature. Nurse data were collected in 2015, and fanner data and tent bee data were collected in 2022. All statistical analyses were performed in R (v4.2.2) and RStudio (v2023.03.0 + 386).

## Results

Overall, we found significant differences in tent bees during the winter compared to their summer counterparts.

We tested the ability of wintering tent bees to learn odors paired with a sucrose reward. We compared this to foraging bees that were collected the summer before. We found that overall, tent bees had a diminished learning response compared to bees collected from the tent (Logistic regression, χ^2^ = 45.498, *P* < 0.0001; Fig. 4).

We also tested the fanning response in tent bees, outdoor fanners, and outdoor nurse bees. We found that the probability of fanning is significantly different across collected groups (Logistic regression: χ^2^ = 39;03, df = 2, *P* < 0.001; Fig. 5) The probability of fanning decreased for inside tent bees compared to colonies maintained outside with fanners collected during the summer(Tukey post hoc test *P* < 0.001), and nurses collected from colonies during the summer (Tukey post hoc test *P* < 0.001). The probability of fanning was not significantly different between nurses and fanners collected from outside colonies during the summer (Tukey post hoc test *P* = 0.68).

We found no significant difference in the response thresholds of bees that do fan in tent bees compared to outside bees or nurse bees (linear regression: *P* = 0.132).

## Discussion

Overall, we found that our method of keeping small honey bee colonies inside of controlled indoor tents was highly effective at keeping colonies active and accessible throughout a temperate winter.

While this indoor wintering technique did produce usable bees, there were clear differences in PER and fanning behavior in bees from indoor winter colonies compared to those from conventionally-managed, outdoor, summer colonies. There could be multiple explanations for these results, including the environmental differences between the outdoor and indoor environments, the behavior of winter and summer bees, or the age of the bees used in the studies; however, our experiments were not designed to elucidate how or why these differences occurred. Instead, they are meant to simply illustrate that, by whatever mechanism, our indoor tent bees performed very differently in these assays than bees from conventional summer colonies.

We underline the importance of our behavioral results as a major caveat; researchers using these methods must take care about the experimental design and conclusions that can be made using bees kept in this manner. For example, behavioral responses from bees in winter tents may not be representative of the behavior of bees in summer colonies. Despite this limitation, bees produced in these colonies are still extremely useful. For example, they can be used for training purposes, teaching, and piloting small experiments. Students can be trained on many assays, learn bee safety, and still make meaningful progress with these colonies. Some approaches or uses may also be more effective than others. For example, because the colony environment plays a smaller role, these colonies may be useful sources of young larvae to be used for experiments studying in vitro reared larvae (Schmehl et al. 2016). We have also used this approach to produce honey bee pupae as the raw material with which to produce or amplify virus particles for use in later experiments (Hsieh et al. 2020), as this use does not require behavioral responses at all. Producing small numbers of fresh bees could also be highly beneficial for developing and testing materials, for example, RNAi experiments that knock down gene expression (Wang et al. 2013). In addition to research uses, these colonies can provide materials for use in teaching or outreach events. For example, indoor colonies could be manipulated into portable observation hives, allowing their use in university or K-12 classroom settings or in public engagement events, though care must be taken to keep the bees protected during transport by minimizing light and extreme temperature exposure.

As such, this method can significantly increase research productivity by expanding the time period during which live bees are accessible throughout the year. While our work occurred in the Northern United States, honey bees exhibit inaccessibility due to winter weather in most temperate climates. However, our methods may be of use to researchers in other regions, even those without cold winters. For example, even in the tropics, where temperatures comparatively stable and warm, variation in hot or dry conditions can cause seasonal variation in activity and brood rearing (Winston et al. 1983) and produce workers that exhibit winter bee-like physiology (Feliciano-Cardona et al. 2020). Therefore, while the timing of our methods may be quite different, researchers in other regions may benefit from adjusting our approach for the maintenance of colonies in indoor tents outside of traditional “winter” conditions. Further, this method is specifically for extending the use period for honey bee research, teaching, and outreach. This method is not for increasing the survival of colonies. Our very preliminary results using 4 colonies over 2 yr illustrate that colonies can survive, as we have had 100% colony survival. As such, we emphasize this is not the goal.

Overall, while bees kept in indoor tents during the winter may be behaviorally and physiologically different than those normally used during the summer field season, there are several major benefits to keeping and using honey bee colonies in the manner. (i) training new students on honey bee basics, such as collecting bees, protocols, and safety; (ii) utilizing larvae or hive material for nonbehavioral studies, such as research on diseases or genetics; (iii) allowing continuing students to design and pilot future studies; (iv) access to colonies for teaching; (v) access to colonies for outreach events. Extending the bee season has allowed us to use honey bees in the classroom and invite administrators and donors to visit the lab to observe bees, even in the middle of winter. These teachable moments to a broad community are highly beneficial when studying such a charismatic and popular insect. Overall, this is an easy, affordable method with real benefits, and we encourage researchers to adopt this indoor beekeeping method.
